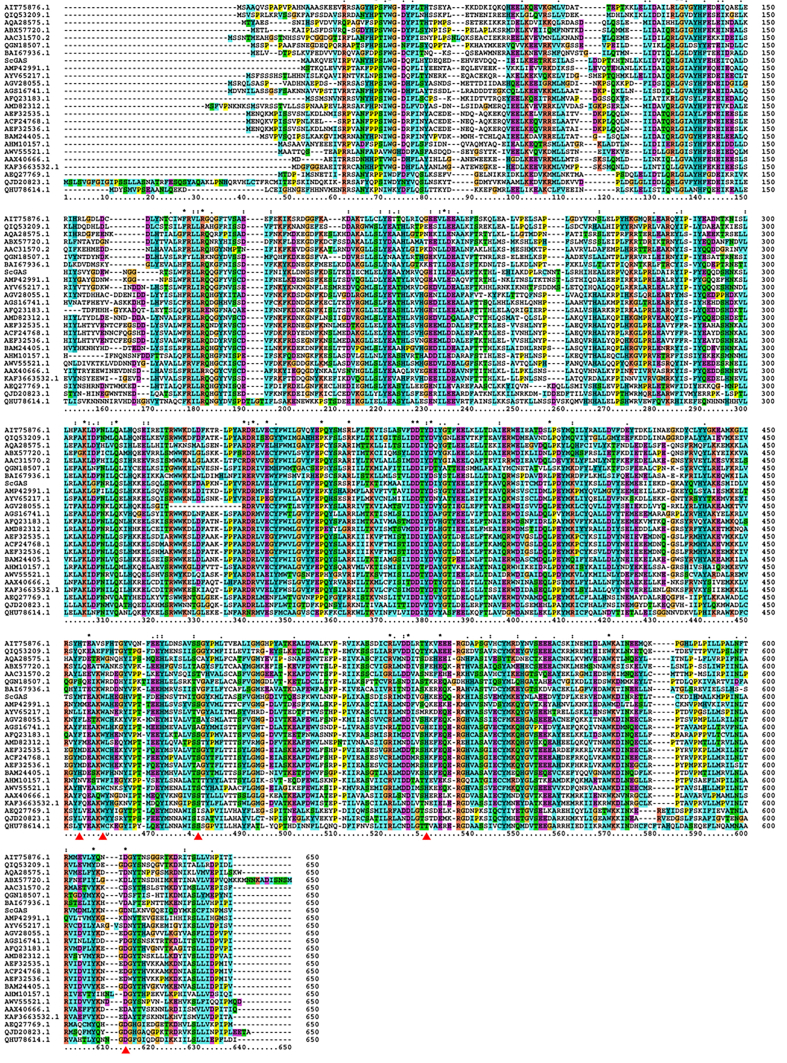# Exploration and mutagenesis of the germacrene A synthase from *Solidago canadensis* to enhance germacrene A production in *E.coli*

**DOI:** 10.1016/j.synbio.2025.02.015

**Published:** 2025-02-28

**Authors:** Jinyan Huo, Xiaohui Chu, Bo Hong, Ruo Lv, Xiaoyu Wang, Jianxu Li, Ge Jiang, Baomin Feng, Zongxia Yu

**Affiliations:** a Jiangxi Key Laboratory for Sustainable Utilization of Chinese Materia Medica Resource, Lushan Botanical Garden, Chinese Academy of Sciences, Jiujiang, 332000, China; b College of Life and Health, Dalian University, Dalian, 116622, China; c Shanghai Key Laboratory of Plant Functional Genomics and Resources, Shanghai Chenshan Botanical Garden, Shanghai Chenshan Plant Science Research Center (CAS), Shanghai, 201602, China

**Keywords:** β-elemene chassis, Germacrene A, Germacrene A synthase, Molecular docking, ScGAS mutagenesis

## Abstract

β-elemene is an effective anti-cancer component which has been widely used in clinic. However, it still relies on the extraction from the Chinese medicine plant *Curcuma wenyujin*, which seriously limits its application. Synthetic biology offers a promising approach to satisfy its supply. β-elemene is derived from germacrene A (GA), which is synthesized by germacrene A synthase (GAS), through Cope rearrangement under heat condition instead of enzymatic reaction. In this study, an effective germacrene A synthase (ScGAS) was identified from *Solidago canadensis* which could produce GA when expressed in *E.coli*. By introducing the heterogeneous MVA pathway to enrich the FPP pool, the strain yielded 147 mg/L of GA in shake flasks which represented 2.98-fold improvement over the initial one. Moreover, combining molecular docking with phylogeny analysis of ScGAS largely narrowed down the category of its key residues' mutagenesis. The Y376L mutant showed the highest yield of 487 mg/L which was almost 10-fold higher than the initial yield. These results indicate that diverting the metabolism of the host and enzyme mutagenesis based on the combination of molecular docking and phylogeny analysis are of great value to constructing terpenoids chassis.

## 1 Introduction

β-elemene, an effective anti-cancer component, has been applied in the treatment of various cancers including lung, liver, brain, breast, ovary, gastric and prostate cancers without causing significant side effects. It plays a crucial role in anti-tumor activities by impeding cell proliferation, arresting cell cycle, promoting cell apoptosis, inhibiting angiogenesis and metastasis, reducing Multiple-Drug resistance (MDR), strengthening the immune system, relieving pain and enhancing radiosensitivity [1]. β-elemene injection has been approved as a non-cytotoxic Class II antitumor drug in China and employed in tumor therapy since 1993. β-elemene is mainly extracted from the traditional Chinese medicine plant named *Curcuma wenyujin* Y. H. Chen et C. Ling [2]. However, limited plant resources and the extremely low concentration of β-elemene pose challenges in meeting the clinical demand. Many efforts have been made to alleviate the shortage of β-elemene, including cell suspension culture, rapid regeneration of special plants with relatively high level of β-elemene, and exploration of β-elemene content in vast plants [[3], [4], [5]]. Nonetheless, the outcomes are not so satisfying. One plausible explanation is that as a secondary metabolite, the synthesis of β-elemene is under tight regulation in plants. Unlike plant extraction, which produces a mixture, chemical synthesis can yield pure β-elemene. However, its application is restricted by the tedious steps, low yield, and environmental pollution. With the rise of metabolic engineering, the synthesis of β-elemene in microbes will make it possible to provide a consistent and alternative resource for the pharmaceutical market.

The extensive history of fermentation and the advent of genetic engineering led to the rise of metabolic engineering in the 1990s, which rewires the metabolism of the host to either enhance the production of native compounds or generate novel metabolites. Metabolic engineering was initially utilized for optimizing microbial fermentation. With new tools like rapid gene synthesis, genome editing and metabolite analysis, it is now employed to tackle the challenge of producing the natural products that are urgently demanded in the market but extremely limited in plants. Metabolic engineering has broad applications in fuels, perfumes and pharmaceutical industries. Microbes like *E.* *coli* and yeast are popular hosts due to their ease of genetic manipulation, scalability and high productivity. Noteworthy achievements include the production of feed additive lysine [6], the chemical building blocks of 1,3-propanediol and 1,4-butanediol [7,8], antibiotic precursor 7-aminodesacetoxycephalosporanic acid (7-ADCA), advanced biofuel isobutanol [[9], [10], [11]], and pharmaceutical drugs like terpenoids [12]. The milestone in the metabolic engineering of terpenoids is the semi-synthesis and industrialization of the anti-malaria sesquiterpene artemisinin. Its precursor artemisinic acid is produced in *Saccharomyces cerevisiae* and then converted into artemisinin by singlet oxygen [13]. The titer of diterpene taxadiene, an intermediate of anti-tumor drug Taxol, reached 1 g/L in an engineered *E.* *coli* through a multivariate-modular approach [14]. Similarly, the titer of miltiradiene, a diterpenoid precursor of anti-inflammatory component tanshionones, achieved 365 mg/L in *S*. *cerevisiae* by combining the modular pathway engineering strategy with systematically assembling terpenoids synthases [15]. These researches provide valuable references for the metabolic engineering of other terpenoids.

β-elemene is a sesquiterpene derived from germacrene A (GA) through Cope rearrangement under heat condition [16]. Terpenoids are composed of C5 blocks known as isopentenyl diphosphate (IPP) and dimethylallyl diphosphate (DMAPP), which combine to form geranyl diphosphate (GPP, C10), farnesyl diphosphate (FPP, C15) and geranylgeranyl diphosphate (GGPP, C20), the precursors of monoterpenes, sesquiterpenes and diterpenes respectively. These C5 blocks are from either the cytoplasmic mevalonate pathway (MVA) or the plastidic 1-deoxy-d-xylulose-5-phosphate pathway (DXP) depending on the organisms [17,18]. Most eukaryotes like yeast use the MVA pathway while prokaryotes like *E.* *coli* can exploit either pathway but prefer DXP. Plants typically utilize the MVA for the synthesis of sesquiterpenes and triterpenes whilst utilizing the DXP for the synthesis of monoterpenes and diterpenes [19]. The premise of successful metabolic engineering is that all enzymes of the pathway of the terpenoid in the native plant are fully elucidated. This can be exemplified by the successful heterogeneous synthesis of artemisinin, in contrast to the hindrance encountered in taxol synthesis. Whereas one determinant of metabolic engineering's success is redirecting the carbon fluxes toward products of interest, the critical challenge is how to ensure the heterologous pathway works well in a non-native host. The hetero-synthesis of sesquiterpene amorpha-4,11-diene in *E.* *coli*, an intermediate of artemisinin, is the first landmark of plant natural product in synthetic biology. The MVA pathway from *S*. *cerevisiae* was first modified by replacing the two HMG-CoA synthases (HMGS, ERG13) and the catalytic domain of HMG-CoA reductase (tHMGR, HMG1) with those from *Staphylococcus aureus*. By incorporating the modified MVA pathway into *E.* *coli* along with fermentation process optimization, the final titer of amorpha-4,11-diene reached more than 25 g/L [20,21].

β-elemene is generated from GA. Efforts to improve β-elemene yield have focused on increasing the precursor GA levels. Over-expressing *tHMGR* in fusion with FPP synthase and GA synthase (GAS) *LTC2* from *Lactuca sativa* led to a six-fold-increase of GA in *S*. *cerevisiae*, reaching 190 mg/L in the shaking flask [22]. Recently *AvGAS* from *Anabaena variabilis* was found to work better than *LTC2* and eight other *GASs*, and reached a higher level of 309 mg/L in the shake-flask culture with *tHMGR1* over-expression, squalene synthesis repression and *AvGAS* mutagenesis [23]. *E.* *coli* is also a robust synthetic platform due to its ease of genetic manipulation and cost-effectiveness compared with yeast, moreover, redirecting carbon fluxes towards specific metabolite in *E.* *coli* is more convenient based on the in-depth knowledge of its metabolic regulation [[24], [25], [26]]. The production of GA in *E.* *coli* is feasible, because GAS just localizes in the cytoplasm eliminating the need for plastids in yeast. In *E.* *coli*, LTC2 showed higher enzyme activities than the other seven GASs, and the yield was further elevated from 75 mg/L to 126 mg/L by an I364K-T410S double mutant [27]. Thus, the activities of GASs from different organisms vary a lot.

In this study, an active ScGAS from *Solidago canadensis* was selected by comparing GAS enzyme activities in the public database and GA content in the extractions of various plants. Then *ScGAS* was co-expressed with a modified heterogeneous MVA pathway to enrich the sesquiterpene precursor FPP pool, then the structure of ScGAS was simulated, docked with FPP, and the key amino acids were mutated to improve GA titer. Ultimately, the titer of GA reached a level 10 times higher than the initial amount.

## 2 Materials and methods

### 2.1 Vector construction

**Fig. 1:**
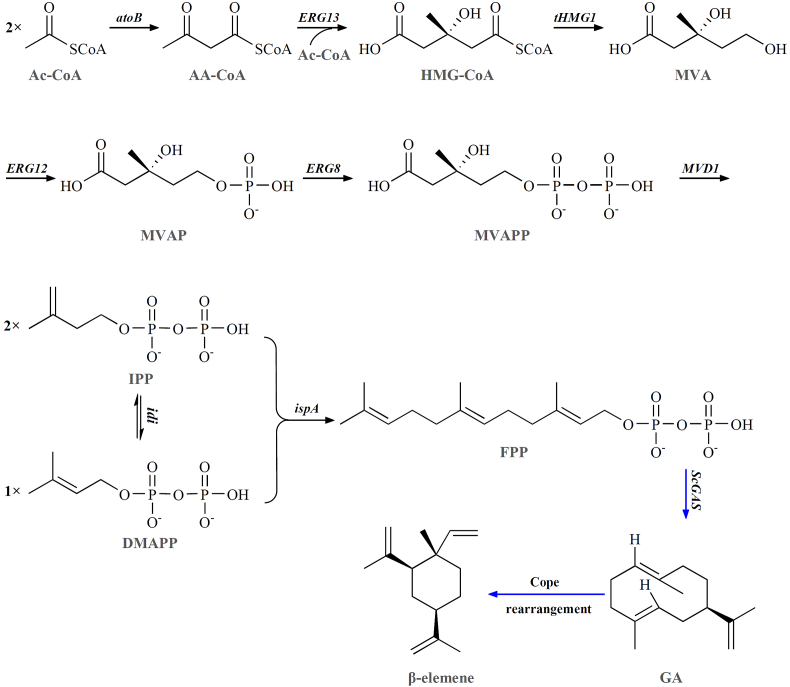
Schedule of the engineered pathway to produce germacrene A (GA) in *E. coli*. The black arrows indicate the strengthened MVA pathway to enrich the FPP pool, which is constructed as pFPP plasmid. The blue arrows show GA, the precursor of β-elemene, mediating the flow from FPP to the final product. Gene list: atoB, acetoacetyl-CoA thiolase; ERG13, HMG-CoA synthase; tHMG1, truncated HMG-CoA reductase; ERG12, mevalonate kinase; ERG8, phosphomevalonate kinase; MVD1, mevalonate pyrophosphate decarboxylase; idi, IPP isomerase; ispA, farnesyl pyrophosphate synthase; ScGAS, germacrene A synthase from *Solidago canadensis*. Intermediates: Ac-CoA, acetyl-CoA; AA-CoA, acetoacetyl-CoA; HMG-CoA, hydroxymethylglutaryl-CoA; MVAP, mevalonate 5-phosphate; MVAPP, mevalonate pyrophosphate; IPP, isopentenyl pyrophosphate; DMAPP, dimethylallyl pyrophosphate; FPP, farnesyl pyrophosphate; Deriviation: *atoB*, *idi*, *ispA* are from *E. coli*, the rest genes except for *ScGAS* are from *S*. *cerevisiae*.

*ScGAS* (GenBanK accession number: AJ304452.1) was codon optimized. The mutants of *ScGAS* were constructed by using the Site-directed Mutagenesis Kit (Sangon). Then both the optimized wild type (WT) and mutants of *ScGAS* were constructed into the plasmid pGEX-4T-1 to form the WT, Y376L, W382R, G402C, H451A, H451L, H451W, D526P vectors in brief. These plasmids expressing *ScGAS* or its mutants were co-transformed with pFPP or pACYCDuet-1 (the backbone of pFPP, as control) to gain the recombinant strains. pFPP is the artificial pathway to strengthen FPP pool including enzymes genes of *atoB*, *ERG13*, *tHMG1*, *ERG12*, *ERG8*, *MVD1*, *idi* and *ispA* (Fig. 1).

### 2.2 Plant essential oil extraction

∼100 mg fresh plant leaves were weighted and frozen in liquid nitrogen, ground into powder, mixed with 1 mL extraction buffer (Ethyl acetate and hexane with a volume ratio 15:85, 20 ng/μL nonyl acetate was added as the internal standard) and sonicated for 20 min, then centrifuged at room temperature, the supernatant was dehydrated with anhydrous Na_2_SO_4_ to finally obtain the plant essential oil.

### 2.3 Culture conditions and product extraction

The overnight liquid cultures of strains were inoculated into LB medium containing proper antibiotics at the ratio of 1:50. When OD_600_ reached around 0.5, the culture was chilled on ice and added IPTG (final concentration was 0.5 mM) and dodecane (20 % of the total culture volume), and shaken in the 28 °C incubator at 200 rpm for 48 h. For optimizing the culture conditions, the parameters were set according to the L_9_ (3^4^) orthogonal experiment table.

To extract the product, the culture was chilled to room temperature, the same volume of ethyl acetate was added and mixed well before sonication for 5 min. After centrifugation, the organic layer was collected for GC-MS analysis.

### 2.4 Volatile detection

The plant essential oils and products of ScGAS recombinant strains were analyzed by GC-MS using HP-5MS (30 m × 0.25 mm × 0.25 μm) column. 1 μL solution was injected in the splitless mode. The injector temperature was 250 °C and the carrier gas was helium at 1 mL/min rate. The program was as follows: 80 °C for 3 min; raised to 150 °C at 10 °C/min and hold for 1 min; heated to 170 °C at 2 °C/min; then raised to 210 °C at 15 °C/min and hold for 1 min. Because GA is fully transformed into β-elemene at high temperature during the GC-MS analysis, β-elemene was used to quantify GA extracted from plants and *E. coli* cultures. 20 ng/μL nonyl acetate was used as the internal standard. Both the peaks of nonyl acetate and β-elemene were identified by comparison with the similarity to the in-house library. And β-elemene was quantified by firstly comparing the peak area of β-elemene to that of nonyl acetate, then multiplying the volume of the extraction, and finally dividing by the fresh weight of the sample.

### 2.5 Alignment and phylogeny analysis

The amino acid sequences of sesquiterpene synthases from different plant species were downloaded from NCBI website (https://www.ncbi.nlm.nih.gov/). Then ScGAS was aligned with those sequences using Clustal X software. Phylogeny tree was constructed using the maximum likelihood algorithm in TBtools software [28]. Branch point bootstrap values were calculated with 1000 replicates and the tree was drawn to scale.

### 2.6 Homologous modeling of ScGAS

The protein crystal structure of α-bisabolol synthase mutant (PDB accession number 4GAX) which showed the highest homology with ScGAS was chosen as the template, and the homologous model of ScGAS was constructed by Discovery Studio 2022. The parameters for modeling were set as follows: the number of models was 100, the optimization level was set to "High", and the rest were set as default. The model with low scores was selected by comprehensively comparing the Probability Density Functions Total Energy (PDF total energy) and the Discrete Optimized Potential Energy Score (DOPE score). The loop refinement and energy minimization of the model were carried out with Ramachandran Plot and Verify 3D. The number of models for loop optimization was set to 5, and the optimization level was selected as "High". For molecular dynamics optimization, the Steepest Descent method and the Conjugate Gradient method were used to perform energy optimization for 200 steps. Based on 5-epi-aristolochene synthase crystal structure (PDB accession number 3M01), the magnesium ions were superposed onto its active site and refined by Standard Dynamics Cascade module under the default mode, and the model with the lowest total energy was chosen for subsequent experiments. Under the CHARMM force field, the Lib Dock module was used to dock the active sites between the ligand FPP and the two conserved metal ion binding motifs ("DDXXD" and "NSE/DTE") to form the ScGAS model.

### 2.7 ScGAS mutagenesis and prediction

ScGAS was analyzed online (https://www.predictprotein.org). Consurf analysis estimates the evolutionary rate of amino acids based on correlation between ScGAS and its homology proteins and mapped to the 3D structure in order to find out the key amino acids. Amino acids with a low evolutionary rate are conserved amino acids. SNAP2 saturation mutagenesis is used to estimate the influence of mutations on ScGAS function. A SNAP2 score higher than 60 indicates that the mutant has a significant impact on the protein function. Calculate Mutation Energy (Stability) and Calculate Mutation Energy (Binding) modules of Discovery Studio were used to calculate the free energy of protein folding and ligand binding after mutation, so as to predict the impact of mutation on enzyme activity. Theoretically, when the thermal stability of the protein and the affinity between the enzyme and the substrate are enhanced, the enzyme activity will be improved. The Build Mutants module of Discovery Studio was selected to rebuild the models of these mutants, which were respectively docked with the substrate, and the lib dock score and conformation of the substrate were analyzed as aforementioned.

## 3 Results

### 3.1 Screen for an effective GAS from various plants

**Fig. 2:**
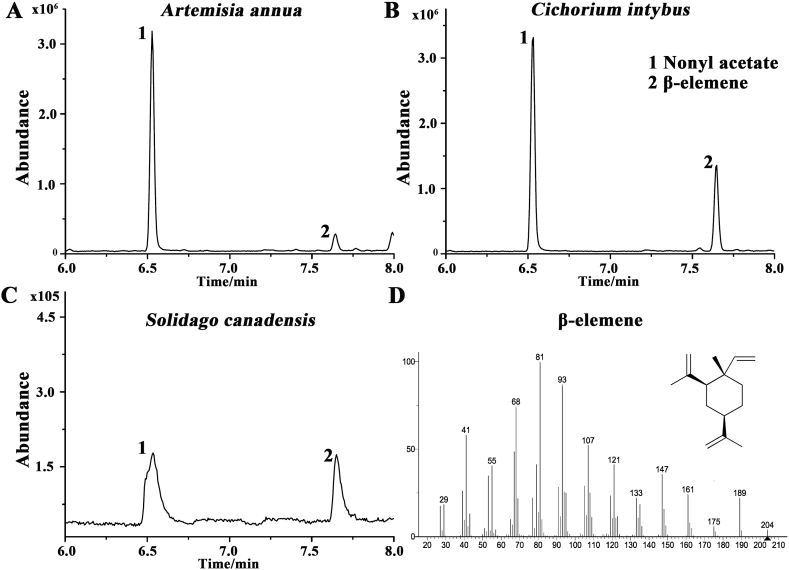
GC-MS detection of GA contents in the leaves of different plants. GC-MS diagrams of *A*. *annua* (A), *C*. *intybus* (B) and *S*. *canadensis* (C). Nonyl acetate was used as the internal standard. (D) Chemical structure and mass spectral fragments of β-elemene, which is used as the indicator of GA contents because GA is completely converted into β-elemene at the high temperature during GC-MS analysis.

**fig S1:**
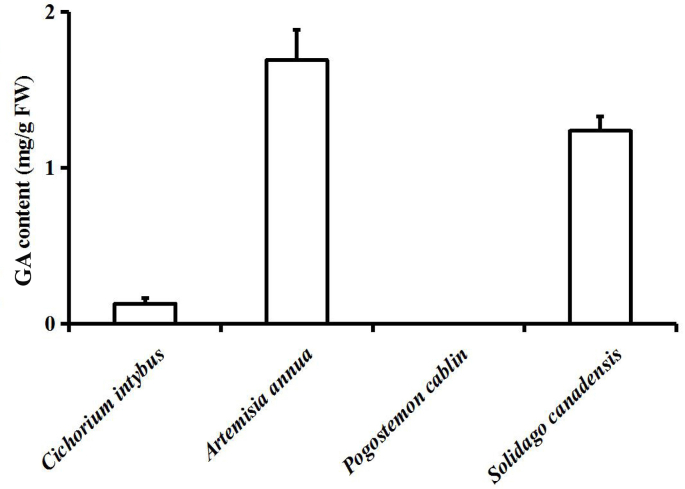


β-elemene is a common component of plant volatile but no β-elemene synthase has been reported so far. Instead, it is generally considered that β-elemene is produced from germacrene A (GA), another ubiquitous plant volatile, by rearrangement. Because GA is fully transformed into β-elemene at high temperature during the GC-MS analysis, β-elemene was used to quantify GA extracted from plants and *E. coli* cultures. Numerous GA synthases (GAS) have been documented in the public database. In this study, we first compared their documented enzyme activities (Supplementary Table 1), as enzyme activity is directly related to the product content, the plants with high, medium and low GAS activities or GA yields were chosen, such as *Artemisia annua*, *Cichorium intybus*, *S*. *canadensis* and *Pogostemon cablin*. We then measured the GA content in the leaves of these plants. The results showed higher levels of GA in *A*. *annua* and *S*. *canadensis*, while it was undetectable in *P*. *cablin* (Fig. 2, Supplementary Fig. 1), confirming that the GASs in *A*. *annua* and *S*. *canadensis* functioned properly. As the lower Km value is, the higher affinity between substrate and enzyme is, *ScGAS* from *S*. *canadensis* was selected for the following investigation.

### 3.2 Synthesize GA in *E.coli* and enhance its production by boosting the FPP pool

**Fig. 3:**
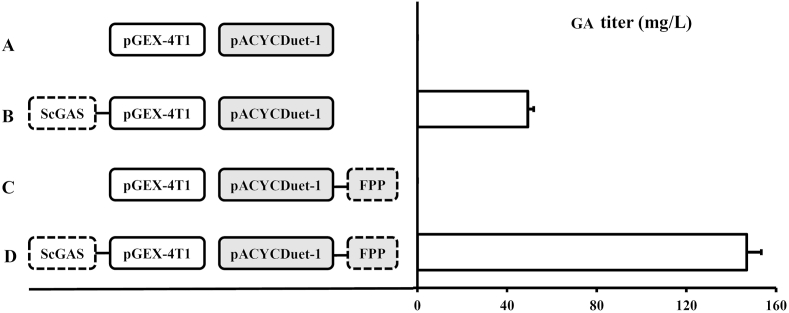
GA titer in recombinant *E.coli*. The diagrams and the titer of recombinant strains are shown on the left and right parts respectively. (A) Negative control with empty vectors. (B) Co-expression of *ScGAS* with the empty vector pACYCDuet-1. (C) Co-expression of the empty vector pGEX-4T1 with pFPP. (D) Co-expression of *ScGAS* with pFPP. Nonyl acetate was used as the internal standard.

GAS transforms its precursor FPP into GA directly, and there is no signal peptide in *ScGAS* (Supplementary file 1), making *E.* *coli* an ideal host for GA biosynthesis. *ScGAS* was codon optimized (Supplementary file 2) and transformed into *E.* *coli*. The initial GA content was 49 mg/L in the shake-flask batch culture (Fig. 3B), because *E.* *coli* possesses a native MEP pathway which ensures a basic FPP level. To increase GA production, the heterogenous MVA pathway (Referred as pFPP, Fig. 1) was constructed and co-expressed with *ScGAS*, the titer reached 147 mg/L (Fig. 3D) which was 2.98 times higher than that of *ScGAS* alone (Fig. 3B). This result showed that redirecting the metabolic flux of the host to enrich the precursor FPP pool was an effective way to boost the production of GA.

### 3.3 Mutagenesis of ScGAS based on the combination of phylogeny analysis and molecular modeling

**Fig. 4:**
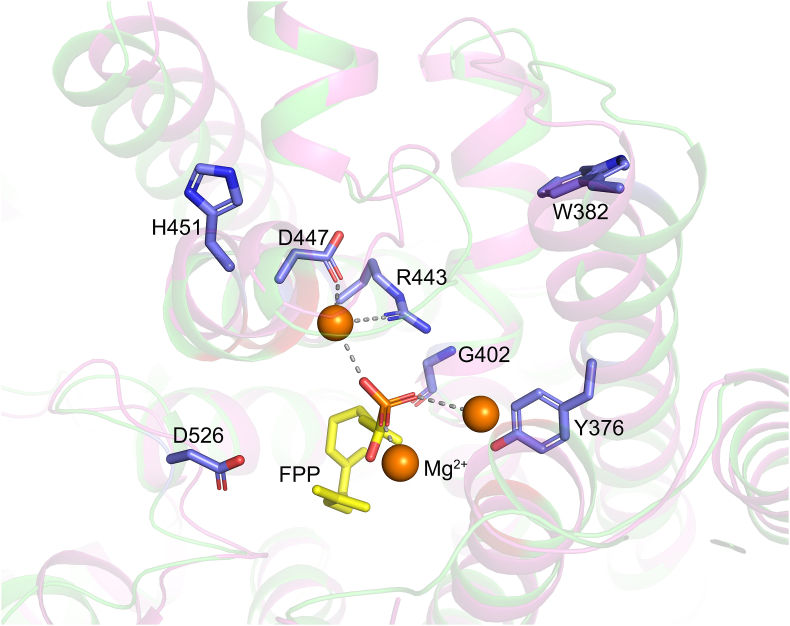
Homology modeling of ScGAS and docking with FPP. The key residues, Mg^2+^ ions and the FPP substrate were shown.

**fig S2:**
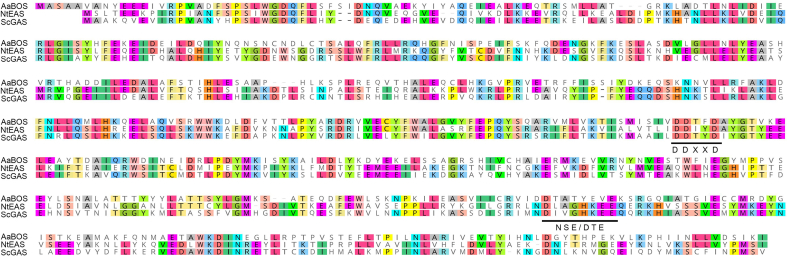


Plasticity residues usually contribute significantly to the enzyme activity. We then looked for the key residues by combination of phylogeny analysis and molecular docking. The three-dimensional structure of ScGAS was first constructed according to homology modeling. ScGAS shows 53.0 % amino acid sequence homology with the bisabolol synthase variant of *A*. *annua* (AaBOS, PDB ID: 4GAX) whose crystal structure was used as a template (Supplementary Fig. 2) [29]. The model with the smallest PDF value was chosen for further loop optimization and energy minimization. Finally, the Mg^2+^ ions were introduced into the ScGAS model and optimized based on the crystal structure of sesquiterpene synthase 5-epi-aristolochene synthase in tobacco (NtEAS, PDB ID: 3M01) [30] (Supplementary Fig. 2). When the substrate FPP interacts with the two Mg^2+^ to form a "U"-shaped conformation, 1,10-cyclization is more likely to occur to generate GA. FPP was docked to the two conserved metal-ion binding motifs ("DDXXD" and "NSE/DTE") in the "U" configuration (Fig. 4), and the amino acids that affected the docking effect could be the critical ones for enzyme activity.

24 plasticity residues that are nearby or right in the conserved DDXXD and NSE/DTE motifs or the catalytic pocket could potentially impact the docking of ScGAS with FPP. These residues include Y15, R264, W273, A298, D301, D302, D305, Y364, Y376, E379, W382, H394, T398, T401, G402, N446, H451, E454, Q455, R457, Y522, D526, L528 and K529. Saturation mutations of these residues were employed and estimated by comprehensively comparing the SNAP2 score, the protein stability and binding affinity to the substrate as well as the optimum binding conformation of FPP (Supplementary Table 2), the key residues were narrowed down to five as follows Y376, W382, G402, H451, D526. Which amino acid should the residue be mutate to? Considering that there are 19 other common amino acids, possibilities were still too many to investigate.

**Fig. 5:**
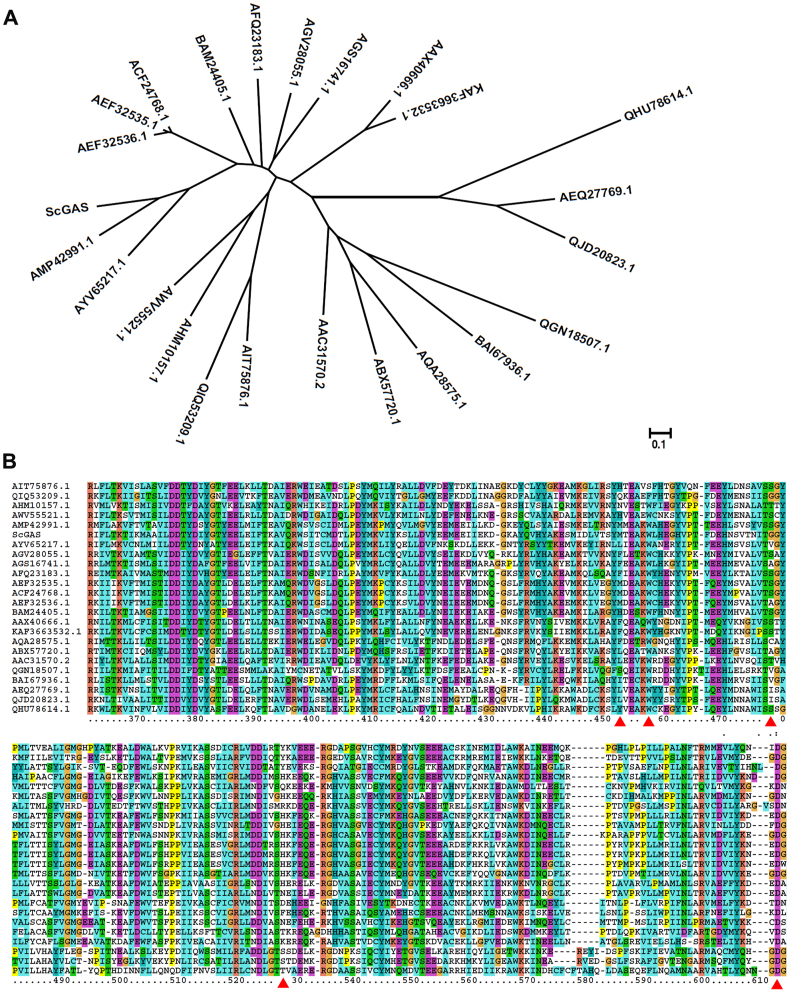
Phylogeny analysis of ScGAS with other sesquiterpene synthases. (A) Phylogeny tree of ScGAS with other sesquiterpene synthases. The maximum likelihood tree was constructed by TBtools. Branch point bootstrap values were calculated with 1000 replicates, and the tree was drawn to scale. (B) Align the protein sequences of ScGAS with other sesquiterpene synthases using Clustal X. The region of the key residues was shown. The whole alignment result was given as Supplementary Fig. 3. The red triangles indicate the key amino acid residues.

**Fig. 6:**
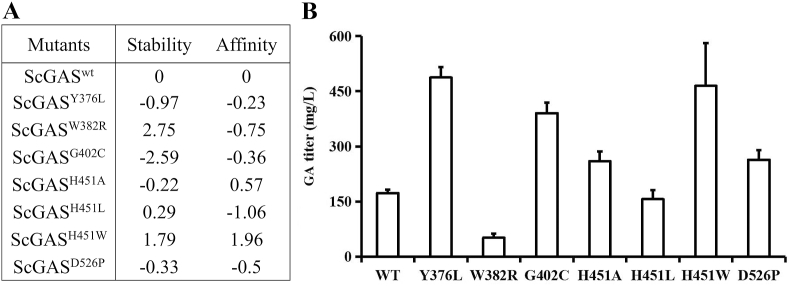
Stability, affinity (A) and enzyme activity (B) after residues' mutations in ScGAS.

Phylogeny analysis was employed. Y376, W382 and D526 were all conserved among GASs (Supplementary Fig. 4). Then the analysis was expanded to sesquiterpene synthases, and only D526 was still conserved (Fig. 5). The natural variants of certain residues were considered first like Y376L (Fig. 5). But when there were too many variants, the saturation mutations (Supplementary Table 2) were fully considered. To evaluate the efficiency of saturation mutations, the mutants with maximum, moderate or limited effects on the docking were selected for further enzyme activity analysis including Y376L, W382R, G402C, H451A, H451L, H451W and D526P. H451 lies right in the NSE/DTE domain indicating its key roles in ScGAS activity, three mutants with different estimated effects were tested (Fig. 6A). The activities of all mutants were increased by strengthening either protein stability or binding affinity to different extend, with the highest yield of Y376L reaching 487 mg/L (Fig. 6). Thus, as predicted, enhancing the protein stability or binding affinity of key residues surrounding the pocket could optimize the enzyme activity. The exceptions were W382R and H451W. The plausible explanation for W382R was sacrificing the stability leading to the compromise of enzyme activity, but when both the affinity and the stability decreased in H451W, other factors like the size and charges of the pocket may determine the activities.

### 3.4 Optimization the culture conditions

**Fig. 7:**
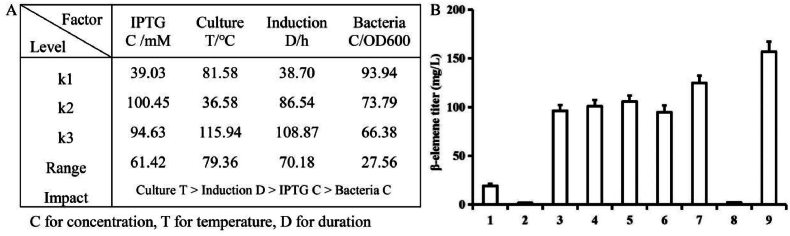
Range analysis (A) and GA detection (B) of different combinations in the orthogonal experiment. The culture conditions of No.1–9 in (B) are listed in Supplementary Table 4.

To further improve the titer of GA, the main factors affecting the titer, such as bacteria concentration, IPTG concentration, culture temperature and the duration of induction were investigated. Three different levels were set for each factor as shown in Supplementary Table 3. We then performed the L_9_ (3^4^) orthogonal experiment (Supplementary Table 4). A range analysis was conducted to assess the influences of these factors on GA production in the recombinant *E.* *coli* co-expressing pFPP and *ScGAS*. The results demonstrated that the culture temperature was the primary impact factor followed by the induction duration and IPTG concentration, whereas the bacteria concentration showed the minimum effect (Fig. 7A). The maximum yield came from group 9th with a titer of 157 mg/L (Supplementary Table 4, Fig. 7B). Combining the range analysis with the titers of different groups, the optimum parameters for GA production were determined to be adding 0.1 mM IPTG when the bacteria concentration reached 0.5 (OD_600_) and culturing at 28 °C for 72 h, but they only increased the titer a little.

## 4 Discussion

Synthetic biology is a powerful tool to relieve the limitations of scarce natural products as long as their biosynthesis pathways have been elucidated. There are many strategies to improve the output of engineered strains. For example, optimizing the culture conditions, heterogeneously over-expressing the synthetic pathway and redirecting the carbon flux to enrich the precursors are commonly used. Take the production of germacrene A (GA) as an example, the initial yield of *LTC2*, a germacrene A synthase (GAS) from *L*. *sativa*, was 30 mg/L in *S. cerevisiae*. Further over-expressing *tHMG1* (Truncated hydroxy-3-methylglutaryl coenzyme A reductase, a key enzyme in mevalonate pathway) or fusing farnesyl diphosphate synthase (*FPPS*) with *LTC2* resulted in a 1.5-fold and 3-fold enhancement in the output respectively. Combining *tHMG1* over-expression with *FPPS-LTC2* fusion finally led to a remarkable 6-fold elevation [22]. Host strains and culture media affect the product yield too. The group of BL21(DE3)-MM in SBMSN medium outperformed other combinations of LB, YM9 medium and BL21(DE3) Star, BW25113, BL21 trxB (DE3) strains in producing the precursor FPP [27]. We introduced a heterologous MVA pathway (pFPP) to enhance the amount of FPP. As a result, the titer of GA was amplified about three times, and it was further improved by modifying the culture conditions.

In addition to these classic strategies, selection of the high-effective enzyme is crucial for maximizing the production of desired compounds. Many GASs have been screened to identify enzymes for enhancing the production of GA, including those derived from plants like *Achillea millefolium* (AmGAS), *Taraxacum ofcinale* (ToGAS), *L*. *sativa* (LsGAS, LTC2) and those from microbes like *Nostoc* sp. PCC 7120 (NsGAS), *Nostoc carneum* NIES-2107 (NcGAS), *Calothrix* sp. NIES-2100 (CsGAS), *Methyloglobulus morosus* KoM1 (MmGAS), *Streptomyces platensis* (SpGAS), *Planomonospora sphaerica* (PsGAS) and AvGAS, but only NcGAS, NsGAS and AvGAS conferred the production of GA in *S*. *cerevisiae* with AvGAS as the outstanding one [23], indicating that the original activities of GASs from different species vary a lot. Therefore, the identification of a high-efficiency enzyme via high-throughput screening is fundamental to creating a robust cell factory. Typically, phylogeny analysis serves as the initial step for enzyme screening, but it should be noted that sequence identity does not guarantee the similar activities. In our case, the product of GASs, GA, was double checked to confirm that the enzymes functioned properly.

Site-directed mutagenesis is commonly the next approach to improve the enzyme activity; however, it is intractable to get a valid mutant. Most former cases were "mutants to death" to verify the crucial residues in maintaining the enzyme activity, but "mutants to vibrancy" is what really needed in synthetic biology. Previous reports have shed light on the key residues for the sesquiterpene synthase modification. Classic motifs named "DDXXD" and "NSE/DTE" are responsible for enzyme specificity and activity, mutations of the conserved residues in these domains usually abolish or reduce the activities [31,32]. H451 is in the "NSE/DTE" domain of ScGAS, H451L, H451A and H451W mutants lift its yield higher and higher. H is basic while L, A, W are all neutral residues, these mutants introduce a significant charge variation in the pocket. On the other hand, L and A are much smaller than H while W is bigger, this may alter the pocket size and finally the output. Furthermore, the improvement in the final titer may not only rely on the increase on enzyme activity but also the removal of the feedback inhibition. In addition, altering residues proximal to the NSE/DTE domain, which is known as ^pre^NSE/DTE, impacts the promiscuity and fidelity of 1,10/1,11-cyclization of FPP. Substituting the ^pre^NSE/DTE in PatPTSiso (a variant of the sesquiterpene patchoulol synthase in *P*. *cablin*) from A to S could nearly double the proportion of patchoulol [33], which could be employed to increase product the purity of the promiscuous sesquiterpene synthases. Nowadays, many software programs or artificial intelligence tools can assist in predicting the effect of mutagenesis on the enzyme activity, in our case we used Discovery studio for virtual mutagenesis and phylogeny analysis to screen for the key residues and the amino acids they should be mutated to, which largely narrowed the screen scope and indicated the mutant direction. Thus, combining phylogeny analysis with molecular docking will offer more precise insights for enzyme engineering in the near future.

*S. cerevisiae* and *E. coli* are commonly used in synthetic biology. Could other hosts be more productive than them? Recently, many attempts have been made to discover the unconventional hosts. *Ogataea polymorpha* is famous for its heat tolerance and high-density fermentation, the content of GA from *LTC2* in *O. polymorpha* was elevated from 19 mg/L to 509 mg/L in flask and reached 4.7 g/L in fermentation by optimizing the MVA pathway, suppressing the competitive pathways, and increasing NADPH and acetyl-CoA supplements [34]. Similarly, oleaginous yeast *Yarrowia lipolytica* could produce the liposoluble terpenes promising itself as an attractive host for terpene biosynthesis. By reprogramming the metabolism of *Y. lipolytica*, the yield of *dlGAS* from *Daldinia loculata* elevated from around 73 mg/L to 2.8 g/L in flask, which further reached 39 g/L after fermentation optimization [35]. Although plants offer the advantage of photosynthesis, their utilization in synthetic biology is seriously hindered by strict regulatory constraints, unexpected intermediate modification and difficult transgenesis. Anne Osbourn's lab has achieved the production of saponin adjuvants intermediates, a type of triterpenoids, by transiently expressing in *Nicotiana benthamiana* at a preparative scale [36]. This opens up the access to enhance terpenoids production in a plant-based system. Our study provided an excellent enzyme ScGAS, which is expected to perform outstandingly in the fermentation of unconventional hosts.

## 5 Conclusion

We identified an effective germacrene A synthase, ScGAS, through screening enzyme activities and GA levels in diverse plants. Enriching the precursors pool by introducing a heterogeneous MVA pathway coupled with improving ScGAS activities via molecular docking and site-directed mutagenesis led to a substantial increase in the flask titer and reached approximately 10-fold improvement. Other optimization strategies like rewiring the metabolism, screening the hosts and fermentation would probably further elevate the yield. Our results highlight ScGAS as an attractive candidate for GA production, and provide valuable insights for selecting effective enzymes and enhancing their efficiency.

## CRediT authorship contribution statement

**Jinyan Huo:** Visualization, Validation, Software, Methodology, Investigation, Formal analysis, Data curation. **Xiaohui Chu:** Visualization, Validation, Software, Methodology, Investigation, Formal analysis, Data curation. **Bo Hong:** Software, Methodology, Investigation, Data curation. **Ruo Lv:** Software, Methodology, Investigation, Data curation. **Xiaoyu Wang:** Software, Methodology, Investigation, Data curation. **Jianxu Li:** Software, Methodology, Investigation. **Ge Jiang:** Software, Methodology, Investigation. **Baomin Feng:** Funding acquisition, Conceptualization. **Zongxia Yu:** Writing – review & editing, Supervision, Funding acquisition.

## Funding

Z.X. Yu acknowledges the 10.13039/501100001809National Natural Science Foundation of China (No. 32360111), Science Fund for Distinguished Young Scholars of Jiangxi Province (No. 20224ACB215004), 10.13039/100022957Double Thousand Plan of Jiangxi Province (No. jxsq2023101106), 10.13039/501100013064Key Research and Development Program of Jiangxi Province (No. 20243BBI91010) and Open Project Program of 10.13039/501100021157Shanghai Key Laboratory of Plant Functional Genomics and Resources (No. PFGR202303). B.M. Feng acknowledges the Key Research and Development Program of Dalian (No. 2021YF18SN024)

## Declaration of competing interest

The authors declare that they have no known competing financial interests or personal relationships that could have appeared to influence the work reported in this paper.

## Figures and Tables

**fig S3:**