# Supplementary material for: Exploration and mutagenesis of the germacrene A synthase from Solidago canadensis to enhance germacrene A production in E.coli
Source: Synth Syst Biotechnol. 2025 Feb 28;10(2):620–8. doi: 10.1016/j.synbio.2025.02.015 (PMC11946497; doi:10.1016/j.synbio.2025.02.015)

Supplementary file 1. Prediction of *ScGAS*’s sublocalization using SignalP (https://services.healthtech.dtu.dk/services/SignalP-6.0/).


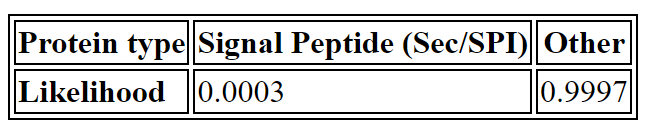


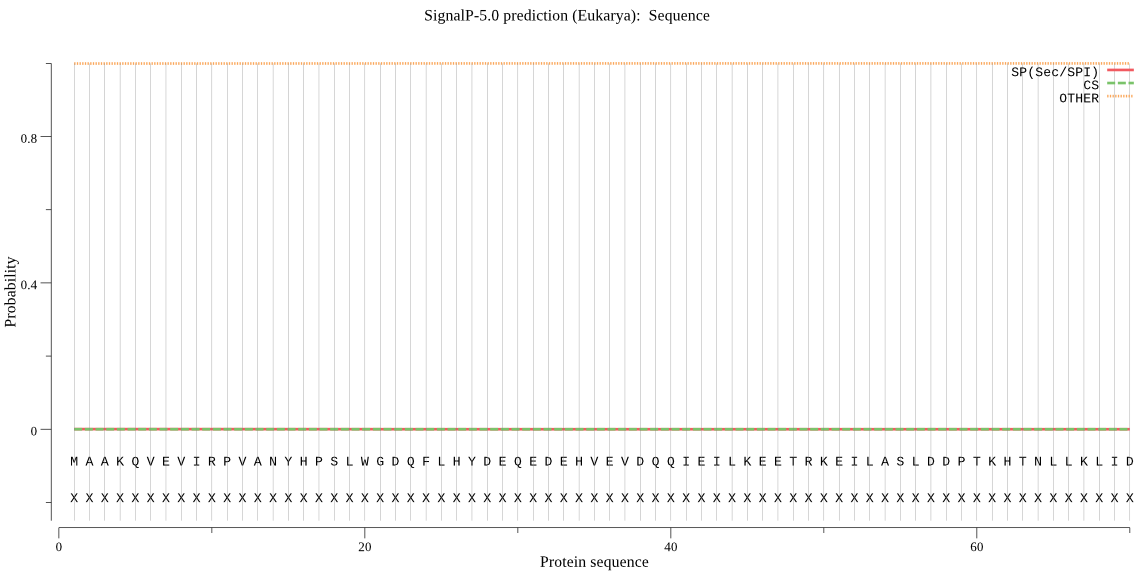

Supplement: Multimedia component 1 [file mmc1.docx]
