# Supplementary material for: Exploration and mutagenesis of the germacrene A synthase from Solidago canadensis to enhance germacrene A production in E.coli
Source: Synth Syst Biotechnol. 2025 Feb 28;10(2):620–8. doi: 10.1016/j.synbio.2025.02.015 (PMC11946497; doi:10.1016/j.synbio.2025.02.015)
Supplement: Multimedia component 2 [file mmc2.docx]

Supplementary file 2. Optimized *ScGAS* sequence according to *E.coli* codon

ATGGCCGCCAAACAGGTTGAAGTGATCCGCCCGGTGGCAAATTATCATCCGAGCCTGTGGGGCGATCAGTTTCTGCATTATGATGAACAGGAAGATGAACATGTTGAAGTTGATCAGCAGATTGAAATCCTGAAAGAAGAAACCCGTAAAGAAATTCTGGCCTCACTGGATGATCCGACCAAACATACGAACCTGCTGAAACTGATTGATGTGATTCAGCGTCTGGGAATTGCTTATTATTTTGAACATGAAATTACCCAGGCACTGGATCATATTTATAGTGTTTATGGTGATGAATGGAATGGTGGTCGTACCTCACTGTGGTTTCGTCTGCTGCGTCAGCAGGGCTTTTATGTTTCTTGTGATATTTTTAATATCTATAAACTGGATAATGGTTCTTTTAAAGATAGTCTGACTAAGGATATTGAATGCATGCTGGAACTGTATGAAGCAGCGTATATGCGTGTACAGGGGGAAATTATTCTGGATGAGGCGCTGGAATTTACAAAAACCCATCTGGAACATATTGCCAAAGATCCGCTGCGCTGTAATAATACGCTGAGTCGCCATATTCATGAAGCACTGGAACGTCCGGTGCAGAAACGTCTGCCACGTCTGGATGCCATTCGTTATATTCCGTTTTATGAACAGCAGGATTCTCATAATAAAAGCCTGCTGCGTCTGGCAAAACTGGGTTTTAATCGTCTGCAGTCTCTGCATAAAAAAGAGCTGAGCCAGCTGAGTAAATGGTGGAAAGAATTTGATGCCCCAAAAAATCTGCCATATGTTCGTGATCGCCTGGTGGAACTGTATTTTTGGATTCTGGGTGTTTATTTTGAACCACAGTATAGCCGCAGTCGTATTTTTCTGACCAAAACCATTAAAATGGCCGCCATTCTGGATGATACATATGATATCTATGGCACTTATGAAGAACTGGAAATTTTTACAAAAGCAGTGCAGCGTTGGTCGATTACTTGTATGGATACACTGCCGGATTATATGAAAGTTATTTATAAATCCCTTTTAGATGTGTATGAAGAAATGGAAGAAATTATAGAAAAAGATGGCAAAGCCTATCAGGTTCATTATGCTAAAGAATCAATGATTGATCTGGTTACTAGTTATATGACTGAAGCGAAATGGCTGCATGAAGGCCATGTTCCGACCTTTGATGAACATAATAGCGTGACGAATATTACAGGCGGTTATAAAATGCTGACCGCGAGCAGTTTTGTCGGTATGCATGGTGATATTGTTACCCAGGAAAGTTTTAAATGGGTGCTGAATAACCCGCCGCTGATTAAAGCGAGCAGCGATATTTCACGCATTATGAATGATATTGTTGGTCATAAAGAAGAACAGCAGCGTAAACATATTGCAAGCAGTGTTGAAATGTATATGAAAGAATATAATCTGGCTGAAGAAGATGTTTATGATTTTCTGAAAGAACGCGTTGAAGATGCATGGAAAGATATTAATCGTGAAACCCTGACCTGTAAAGATATTCATATGGCTCTGAAAATGCCGCCGATTAATCTGGCACGTGTTATGGATATGCTGTATAAAAATGGTGATAATCTGAAAAACGTGGGTCAGGAAATACAGGATTATATGAAAAGCTGCTTTATTAATCCGATGAGTGTTTAA
