# Supplementary material for: Exploration and mutagenesis of the germacrene A synthase from Solidago canadensis to enhance germacrene A production in E.coli
Source: Synth Syst Biotechnol. 2025 Feb 28;10(2):620–8. doi: 10.1016/j.synbio.2025.02.015 (PMC11946497; doi:10.1016/j.synbio.2025.02.015)
Supplement: Multimedia component 3 [file mmc3.docx]

**Supplementary table 1. GASs’ kinetic parameters and product’s contents from public database**

| **GenBank ID** | **Enzyme** | **Species** | **Km/μM** | **Kcat/S^-1^** | **GA contents** |
| --- | --- | --- | --- | --- | --- |
| AF497999 [[28](#_ENREF_28)] | CiGASlo | *Cichorium intybus* | 6.9 | - | - |
| AF498000 [[28](#_ENREF_28)] | CiGASsh | *Cichorium intybus* | 3.2 | - | - |
| DQ447636 [[29](#_ENREF_29)] | AaGAS | *Artemisia annua* | 10.8 | 0.25 |  |
| AY508728 [[30](#_ENREF_30)] | PatTpsCF2 | *Pogostemon cablin* | - | - | 1% |
| AJ304452 [[31](#_ENREF_31)] | ScGAS | *Solidago canadensis* | 2.5 | - | 7% |
| AF489965 [[32](#_ENREF_32)] | LsGAS2 | *Lactuca Situua* | 10.9 | 0.28 | - |
| KM066976 [[33](#_ENREF_33)] | BsGAS1 | *Barnadesia spinosa* | 7.8 | 0.13 | - |
| KM066977 [[33](#_ENREF_33)] | BsGAS2 | *Barnadesia spinosa* | 14.8 | 0.28 | - |
| DQ016667 [[34](#_ENREF_34)] | HaGAS1 | *Helianthus annuus* | 0.82 | - | - |
| EU327785 [[34](#_ENREF_34)] | HaGAS2 | *Helianthus annuus* | 0.74 | - | - |

Abbreviations: “-”, study did not mention.
