# Supplementary material for: Exploration and mutagenesis of the germacrene A synthase from Solidago canadensis to enhance germacrene A production in E.coli
Source: Synth Syst Biotechnol. 2025 Feb 28;10(2):620–8. doi: 10.1016/j.synbio.2025.02.015 (PMC11946497; doi:10.1016/j.synbio.2025.02.015)
Supplement: Multimedia component 5 [file mmc5.docx]

Supplementary table 1. The tested parameters of the four factors in bacteria culturing

| Level Factor | IPTG concentration /mM | Culture temperature /℃ | Induction duration /h | Bacteria concentration /OD600 |
| --- | --- | --- | --- | --- |
| 1 | 0.01 | 20 | 24 | 0.5 |
| 2 | 0.1 | 24 | 48 | 1 |
| 3 | 0.5 | 28 | 72 | 2 |
