# Supplementary material for: Exploration and mutagenesis of the germacrene A synthase from Solidago canadensis to enhance germacrene A production in E.coli
Source: Synth Syst Biotechnol. 2025 Feb 28;10(2):620–8. doi: 10.1016/j.synbio.2025.02.015 (PMC11946497; doi:10.1016/j.synbio.2025.02.015)
Supplement: Multimedia component 6 [file mmc6.docx]

Supplementary table 3. Nine testing groups from the orthogonal array design of the four culturing factors

| Factor  No. | IPTG concentration /mM | Culture temperature /℃ | Induction duration /h | Bacteria concentration /OD600 |
| --- | --- | --- | --- | --- |
| 1 | 0.01 | 20 | 24 | 0.5 |
| 2 | 0.01 | 24 | 48 | 1 |
| 3 | 0.01 | 28 | 72 | 2 |
| 4 | 0.1 | 20 | 48 | 2 |
| 5 | 0.1 | 24 | 72 | 0.5 |
| 6 | 0.1 | 28 | 24 | 1 |
| 7 | 0.5 | 20 | 72 | 1 |
| 8 | 0.5 | 24 | 24 | 2 |
| 9 | 0.5 | 28 | 48 | 0.5 |
